# *α*2-HS Glycoprotein in Plasma Extracellular Vesicles Inhibits the Osteogenic Differentiation of Human Mesenchymal Stromal Cells In Vitro

**DOI:** 10.1155/2019/7246479

**Published:** 2019-02-07

**Authors:** Xiaohua Wu, Mengjun Ma, Peng Wang, Zhongyu Xie, Shan Wang, Hongjun Su, Wen Deng, Pei Feng, Chunyan Su, Jiewen Yang, Jinteng Li, Su'an Tang, Yanfeng Wu, Huiyong Shen

**Affiliations:** ^1^Center for Biotherapy, Sun Yat-sen Memorial Hospital, Sun Yat-sen University, 107 Yan-Jiang West Road, Guangzhou 510120, China; ^2^Department of Orthopedics, Sun Yat-sen Memorial Hospital, Sun Yat-sen University, 107 Yan-Jiang West Road, Guangzhou 510120, China

## Abstract

Extracellular vesicles (Evs) contain diverse functional proteins, mRNAs, miRNAs, and DNA fragments, are secreted by various types of cells, and play important roles in cellular communication. Here, we show for the first time that plasma Evs inhibited the osteogenic differentiation of mesenchymal stromal cells (MSCs) in vitro and the level of inhibition was positively correlated with the plasma Evs concentration. Plasma Evs downregulated the expression of markers such as osteocalcin (OCN), Runt-related transcription factor 2 (Runx2), and Osterix at mRNA levels required for osteogenic differentiation and reduced pSmad1/5/8 levels in MSCs. Furthermore, pSmad1/5/8 levels increased and MSCs underwent normal osteogenic differentiation after Evs-derived *α*2-HS glycoprotein (AHSG) function was inhibited with an anti-AHSG neutralizing antibody. However, the levels of pERK1/2, active *β*-catenin, and HES1 were not significantly altered. Therefore, we propose that as essential components of the extracellular microenvironment of MSCs, plasma Evs are taken up by MSCs and subsequently repress osteogenic differentiation through an AHSG-mediated decrease in pSmad1/5/8 levels. Our work identifies plasma Evs as novel regulators of MSC osteogenic differentiation.

## 1. Introduction

Human bone tissue undergoes the dynamic process of bone reconstruction via continuous bone formation and absorption throughout life. MSCs are multipotent, nonhaematopoietic progenitor cells that are isolated from a variety of tissues [1], and these cells differentiate into osteocytes, chondrocytes, and adipocytes [2, 3]. The proliferation and differentiation of MSCs are affected by their microenvironment. In humans, plasma Evs, which are components of the extracellular microenvironment, modulate the functions of MSCs in multiple ways. Evs are small, spherical, bilayer membrane-bound vesicles, and they are found in various bodily fluids, including plasma, malignant pleural effusions, and urine [4–6]. Evs contain numerous proteins and bioactive lipids that vary depending on the specific donor cell type [7, 8]. Evs are enriched with proteins of the transmembrane 4 superfamily (CD9, CD63, and CD81) and with tumour susceptibility gene 101 (TSG101) [9–11].

Plasma Evs have been reported to contain *α*2-HS glycoprotein (AHSG/fetuin-A), a liver secretory glycoprotein that is present at high levels in the plasma and extracellular matrix [12, 13]. AHSG is a carrier protein with multiple functions. One function of AHSG has consistently been confirmed at the molecular level, in cell culture experiments and in living animals, namely, its ability to bind calcium phosphate and to inhibit mineralization. AHSG prevents calcification by buffering excess matrix mineralization and mediating its clearance in the form of calciprotein particles [14]. Furthermore, in a search for endogenous transforming growth factor-beta (TGF*β*) receptor antagonists, sequence homology was found between TGF*β* receptor type II and AHSG [15]. AHSG blocks osteogenic signalling pathways by binding directly to TGF*β* and with greater affinity to the TGF*β*-related BMPs, thereby inhibiting angiosteosis and the osteogenesis differentiation of MSCs [16].

In our previous study, plasma Evs inhibited the osteogenic differentiation of MSCs in vitro, but the mechanism was unknown. In the present study, the administration of AHSG in plasma Evs inhibited the osteogenic differentiation of MSCs in a dose-dependent manner. Levels of pSmad1/5/8 decreased as the concentration of Evs increased and exhibited a positive correlation with alkaline phosphatase (ALP) levels and alizarin red staining. We concluded that as essential components of the extracellular microenvironment of MSCs, plasma Evs are taken up by MSCs and subsequently repress osteogenic differentiation through an AHSG-mediated decrease in pSmad1/5/8 levels.

## 2. Materials and Methods

### 2.1. Isolation of MSCs

Adult bone marrow was extracted from the posterior superior iliac spine under sterile conditions after obtaining informed consent from healthy donors for the harvest of their tissues for research purposes. Bone marrow mononuclear cells were isolated by Ficoll density centrifugation. Mononuclear cells (1 × 10^6^/mL) were cultured in low-glucose Dulbecco's modified Eagle's medium (L-DMEM, Gibco, New York, NY, USA) containing 10% foetal bovine serum (FBS, Gibco, New York, NY, USA) at 37°C in a humidified air and 5% CO_2_ atmosphere. The medium was replenished every 2 days. MSCs that formed colonies within 10 to 14 days were harvested using 0.25% trypsin/ethylenediaminetetraacetic acid (Gibco, New York, NY, USA) [17]. The expanded MSCs retained the capacity to differentiate into osteocytes, chondrocytes, and adipocytes. MSCs were used at the fourth passage in the subsequent experiments.

### 2.2. Flow Cytometry

MSCs were digested and washed with phosphate-buffered saline (PBS, Boster, Wuhan, China). After incubation with specific antibodies, MSCs were washed again and resuspended in PBS. Flow cytometry was performed using a BD Biosciences Influx Cell Sorter (San Jose, CA, USA). Human phycoerythrin- (PE-) conjugated anti-CD29, fluorescein isothiocyanate- (FITC-) conjugated anti-CD73, FITC-conjugated anti-CD105, FITC-conjugated anti-CD45, PE-conjugated anti-CD14, and PE-conjugated anti-HLA-DR antibodies were used for these experiments (all from BD Biosciences, San Diego, CA, USA).

### 2.3. Analysis of the Trilineage Differentiation Potential of MSCs

#### 2.3.1. Osteogenic Differentiation

MSCs were seeded into six-well plates (1 × 10^5^ cells/well) and cultured in osteogenic differentiation medium (OM) consisting of UltraCULTURE™ medium with 2% Ultroser™ G Evs-free serum, 0.1 *μ*M dexamethasone, 10 mM *β*-glycerol phosphate, and 50 *μ*M ascorbic acid (all from Sigma-Aldrich, St. Louis, MO, USA). The medium was replaced every three days. The total culture duration was 14 days. Cells were stained with alizarin red S (ARS, Aoran, Tianjin, China).

#### 2.3.2. Chondrogenic Differentiation

MSCs (2.5 × 10^5^ cells) were centrifuged at 600 ×*g* for 5 min in a 15 mL conical tubes and then cultured with UltraCULTURE™ medium supplemented with 2% Ultroser™ G Evs-free serum, 1% ITS-Premix (Corning), 50 mg/L ascorbic acid (Sigma-Aldrich, St. Louis, MO, USA), 1 mM sodium pyruvate (Sigma-Aldrich, St. Louis, MO, USA), 100 nM dexamethasone (Sigma-Aldrich, St. Louis, MO, USA), and 10 ng/mL TGF-*β*3 (R&D Systems, Minneapolis, MN, USA). The medium was replaced every three days. The total culture duration was 21 days. Cells were stained with Alcian blue GX.

#### 2.3.3. Adipogenic Differentiation

MSCs were seeded into six-well plates (1 × 10^5^ cells/well). UltraCULTURE™ medium was supplemented with 2% Ultroser™ G Evs-free serum, 1 *μ*M dexamethasone, 10 *μ*g/mL insulin, 0.5 mM 3-isobutyl-1-methylxanthine, and 0.2 mM indomethacin (all from Sigma-Aldrich, St. Louis, MO, USA). After 3 days of induction, the medium was replaced with UltraCULTURE™ containing 10 *μ*g/mL insulin for 1 day and then the medium was replaced with the initial induction medium. After three cycles of media changes, cells were cultured with UltraCULTURE™ containing 10 *μ*g/mL insulin until the 14^th^ day. Cells were stained with Oil Red O after paraformaldehyde fixation.

### 2.4. Extraction, Purification, and Characterization of Plasma Evs

Fifty-four volunteers were recruited as donors of human plasma. Evs were isolated from human plasma samples using the procedure described by Thery et al. [18]. Briefly, 6 mL of plasma was diluted with an equal volume of PBS and centrifuged at 2000 ×*g* for 30 min at 4°C, after which the supernatant was transferred to an ultracentrifuge tube (Beckman Coulter, Brea, CA, USA) and centrifuged at 12,000 ×*g* for 45 min at 4°C, and the resulting supernatant was carefully transferred to a new ultracentrifuge tube and centrifuged at 110,000 ×*g* for 2 h at 4°C. Pellets were resuspended in 10 mL of PBS, and the resulting supernatant was filtered through a 0.22 *μ*m filter (Millipore, Billerica, MA, USA). The flow-through was collected in a fresh ultracentrifuge tube, centrifuged at 110,000 ×*g* for 70 min at 4°C, and washed by centrifugation at 110,000 ×*g* for 70 min at 4°C. Evs were resuspended in PBS and stored at -80°C until use in subsequent studies. 12 samples from 3 batches of Evs were all assessed by transmission electron microscopy (Hitachi Limited, Tokyo, Japan), Nanoparticle Tracking Analysis instrument (Malvern, Worcestershire, UK), and immunoblotting.

### 2.5. Internalization of Evs

For uptake studies, purified Evs were labelled with a PKH26 (Red) kit (Sigma-Aldrich, St. Louis, MO, USA) using the previously reported protocols [19]. Briefly, Evs diluted in PBS were added to 0.5 mL of Diluent C. In parallel, 4 *μ*L of PKH26 dye was added to 0.5 mL of Diluent C, and the mixture was incubated with the Evs solution for 4 min at room temperature. Two millilitres of 0.5% bovine serum albumin (Sigma-Aldrich, St. Louis, MO, USA) in PBS was added to bind the excess dye. The labelled Evs were centrifuged at 110,000 ×*g* for 70 min at 4°C, and the Evs pellet was suspended in PBS and used in uptake experiments. PKH26-labelled Evs were cocultured with MSCs for the indicated times. Then, MSCs were fixed and stained with FITC-labelled phalloidin (Invitrogen, Carlsbad, CA, USA) and DAPI (Invitrogen, Carlsbad, CA, USA). Images were obtained using the confocal laser scanning microscope (Carl Zeiss AG, Oberkochen, Germany).

### 2.6. Osteogenic Differentiation Cultures

MSCs were seeded in 12-well plates at a density of 1.5 × 10^4^ cells/cm^2^ in growth medium (GM) consisting of UltraCULTURE™ medium (Lonza, Basel, Switzerland) and 2% Ultroser™ G Evs-free serum (Pall, Washington, NY, USA). When the culture reached 80% confluence, the medium was replaced with OM consisting of UltraCULTURE™ medium with 2% Ultroser™ G Evs-free serum, 0.1 *μ*M dexamethasone, 10 mM *β*-glycerol phosphate, and 50 *μ*M ascorbic acid.

MSCs cultured in GM were designated the control group, MSCs cultured in OM formed the induction group, and MSCs cultured in OM with different concentrations of plasma Evs were designated the Ind+Evs group. The medium was replaced every 3 days for up to 14 days. For the neutralization study, the Ind+Evs group was treated with different antibodies.

### 2.7. Alizarin Red S (ARS) Staining and Quantification

MSCs were fixed with 4% paraformaldehyde (Sigma-Aldrich, St. Louis, MO, USA) and stained with 1% ARS (pH 4.3) (Aoran, Tianjin, China) for 15 min. After 3 washes, the stained cells were observed under a microscope (Olympus, Tokyo, Japan) and photomicrographs were captured. Cells were destained with 10% cetylpyridinium chloride monohydrate (Sigma-Aldrich, St. Louis, MO, USA). A 200 mL aliquot was transferred to a 96-well plate, and the absorbance was measured at 562 nm using a Multimode Reader (Thermo, Waltham, MA, USA).

### 2.8. Measurement of ALP Activity and Staining

ALP activity in MSCs was detected using an ALP activity kit (Jiancheng Biotech, Nanjing, China). Briefly, MSCs were lysed in radioimmunoprecipitation assay lysis buffer (Thermo, Waltham, MA, USA) containing protease inhibitors (Roche, Basel, Switzerland). The lysate was centrifuged at 12,000 revolutions per minute for 30 min at 4°C, and the supernatant was incubated with reaction buffer at 37°C for 15 min. Colour development was terminated with stop solution, and the absorbance was measured at 405 nm. The protein concentration in the lysate was determined using a Pierce BCA protein assay kit (Thermo, Waltham, MA, USA). ALP activity in the supernatant is presented as units per gram of protein per 15 min.

The ALP staining assay was performed using an ALP staining kit (Sigma-Aldrich, St. Louis, MO, USA). Briefly, cells were fixed with a citrate-acetone-formaldehyde fixative solution and then incubated with the alkaline dye solution for 15 min in the dark. The histochemical detection of ALP was observed under a microscope, and photomicrographs were captured.

### 2.9. Quantitative Real-Time PCR (qPCR)

Total RNA was isolated from MSCs using a TRIzol reagent (Invitrogen, Carlsbad, CA, USA). The cDNA templates were synthesised using a PrimeScript RT reagent kit (TaKaRa, Japan) according to the manufacturer's instructions. qPCR was performed on a Light Cycler 480 Real-Time PCR System (Roche, Basel, Switzerland) using SYBR Premix Ex Taq (TaKaRa, Japan). Changes in the relative expression of each mRNA were assessed using the 2^(−ΔΔct)^ method and normalized to the housekeeping gene GAPDH. Primers were designed to amplify GAPDH (forward, 5′-CACTGCCACCCAGAAGA-3′; reverse, 5′-TCCACGACGGACAC ATT-3′), Runx2 (forward, 5′-TGGTTACTGTCATGGC GGGTA-3′; reverse, 5′-TCTCAGA TCGTTGAACCTTGCTA-3′), OCN (forward, 5′-GGCGC TACCTGTATCAATGG-3′; reverse, 5′-GTGGTCAGCCAACTCGTCA-3′), and Osterix (forward, 5′-CCTCTGCGGGA CTCAACAAC-3′; reverse, 5′-AGCCCATTAGTGCTTGTAAAGG-3′).

### 2.10. Western Blot Analysis

MSCs were lysed, and proteins were quantified as described above. After boiling with sample loading buffer (Beyotime, Shanghai, China), equal amounts of protein were separated on a 10% polyacrylamide gel (Invitrogen, Carlsbad, CA, USA) and subsequently transferred to PVDF membranes (Millipore, Billerica, MA, USA). The PVDF membranes were incubated with the following primary antibodies overnight at 4°C: GAPDH (1 : 3000 dilution, Ca# 97166), Smad1 (1 : 1000 dilution, Ca# 6944), phosphorylated Smad1/5/8 (1 : 1000 dilution, Ca# 13820), Smad2/3 (1 : 1000 dilution, Ca# 8685), phosphorylated Smad2/3 (1 : 1000 dilution, Ca# 8828), ERK1/2 (1 : 1000 dilution, Ca# 4695), phosphorylated ERK1/2 (1 : 1000 dilution, Ca# 4370), *β*-catenin (1 : 1000 dilution, Ca# 8480), active *β*-catenin (1 : 1000 dilution, Ca# 4270), HES1 (1 : 1000 dilution, Ca# 11988) TGF*β*1 (1 : 1000 dilution, Ca# 3711) (all from Cell Signaling Technology, Beverly, MA, USA), CD63 (1 : 1000 dilution, Ca# SAB4301607), CD81 (1 : 500 dilution, Ca# SAB3500454), TSG101 (1 : 1000 dilution, Ca# SAB2702167), ApoB (1 : 1000 dilution, Ca# SAB2104820), and AHSG (1 : 1000 dilution, Ca# SAB1403536), IgG1*κ* (1 : 1000 dilution, Ca# M9269), BMP2 (1 : 1000 dilution, Ca# SAB4301880) (all from Sigma-Aldrich, St. Louis, MO, USA). Membranes were incubated with HRP-conjugated anti-mouse or anti-rabbit secondary antibodies (1 : 3000 dilution; Santa Cruz, CA, USA) for 1 h at room temperature. Specific antibody-antigen complexes were detected using Immobilon Western Chemiluminescent HRP Substrate (Millipore, Billerica, MA, USA).

### 2.11. LC-MS/MS

Evs were lysed, and proteins were quantified as described above. The LC-MS/MS analysis was performed by Guangzhou FitGene Biotechnology Co. Ltd., as previously described [20].

### 2.12. Protein Identification and Data Analysis

The raw files were converted to Mascot generic format (.mgf) files using Proteome Discoverer 1.4 (Thermo, Waltham, MA, USA) with default settings for an in-depth proteome analysis. Protein Pilot 5.0 software (AB Sciex, Foster City, CA, USA) was used for the in-depth proteome analysis and quantitative analysis of proteins with .mgf files as the input. The Paragon algorithm integrated in Protein Pilot 5.0 software was used to search the database.

Briefly, we chose the parameter “Thorough ID” mode with a 95% confidence interval. Only proteins with reasonable ratios across all channels were quantified to increase the confidence level [21–23]. Finally, we identified 571 proteins. A gene ontology (GO) analysis of differentially accumulated proteins was performed using QuickGO software, which utilizes authoritative bioinformatics databases to generate gene symbols for compiled biological processes, molecular functions, and cellular components. The KEGG database (http://www.genome.jp/kegg/pathway.html) was employed to use the current knowledge of biochemical pathways and other types of molecular interactions to examine differentially accumulated proteins. Additionally, STRING 9.1 was used to explore the interaction network and functional relations among the differentially expressed proteins.

### 2.13. Statistical Analyses

Data are presented as mean ± standard errors (SEs). Depending on the type of data, one-way ANOVA or a *t*-test was used for the analysis. Statistical analyses were conducted using SPSS software (SPSS Inc., Chicago, IL, USA). A *p* value of less than 0.05 indicated a significant difference.

## 3. Results

### 3.1. Phenotypic Characterization and Trilineage Differentiation of MSCs

**Figure 1:**
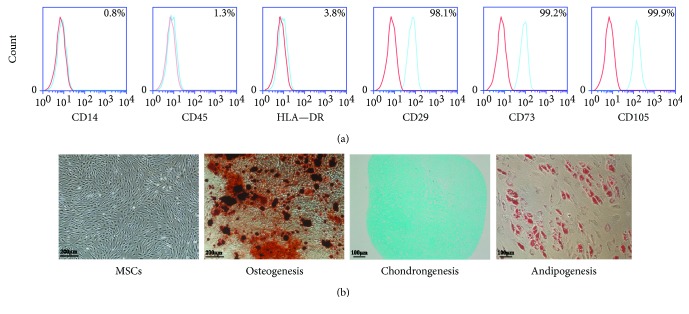
Identification of MSCs. (a) Flow cytometry was used to identify phenotypic surface markers of MSCs. All MSCs from different donors were positive for CD29, CD73, and CD105 but negative for CD14, CD45, and HLA-DR, confirming the typical MSC phenotype. (b) Osteogenic, chondrogenic, and adipogenic differentiation were successfully induced.

Flow cytometry was used to identify the phenotypic surface markers of MSCs. All MSCs from the various donors were positive for CD29, CD73, and CD105 but negative for CD14, CD45, and HLA-DR, confirming a typical MSC phenotype (Figure 1(a)). Osteogenic, chondrogenic, and adipogenic differentiation were successfully induced (Figure 1(b)).

### 3.2. Plasma Evs Are Transferred to MSCs

**Figure 2:**
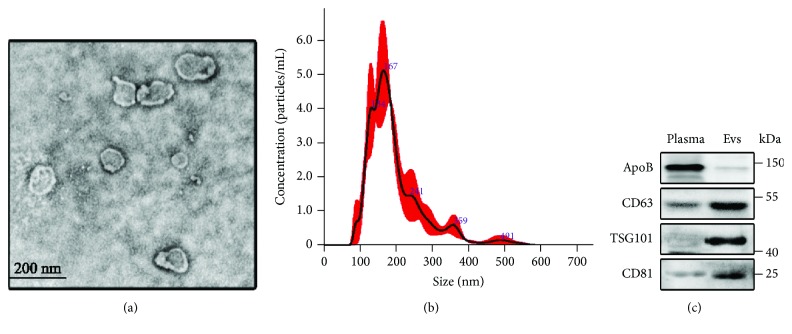
Identification of plasma Evs. (a) Ultrastructure of plasma Evs as determined by transmission electron microscopy; scale bar: 200 nm. (b) Size distribution profile of plasma Evs determined by NanoSight. (c) Analysis of the expression of ApoB, CD63, TSG101, and CD81 by Western blotting.

Numerous characteristics of Evs were examined, including electron microscopic features, particle size, and surface markers. The transmission electron microscopy analysis of plasma Evs revealed the presence of spherical vesicles with a typical cup shape (Figure 2(a)). We detected the size distribution profile of plasma Evs using NanoSight and identified a peak at 167 nm (Figure 2(b)). The isolated Evs expressed CD63, CD81, and TSG101 at high levels, but ApoB at low levels (Figure 2(c)).

**Figure 3:**
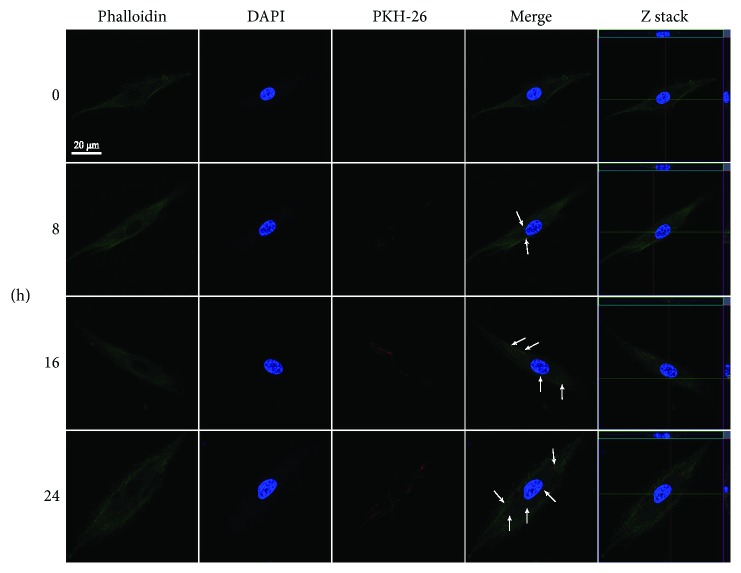
Internalization of the plasma Evs into MSCs. MSCs were stained with phalloidin (green), and the nuclei were counterstained with DAPI (blue). PKH26-labelled plasma Evs (red) were incubated with MSCs.

We next examined whether plasma Evs were transferred to MSCs. After isolation from plasma samples, Evs were fluorescently labelled with PKH26 and incubated with MSCs. We confirmed the ability of MSCs to take up Evs as soon as 8 h after incubation (Figure 3).

### 3.3. Plasma Evs Suppress MSC Osteogenic Differentiation

**Figure 4:**
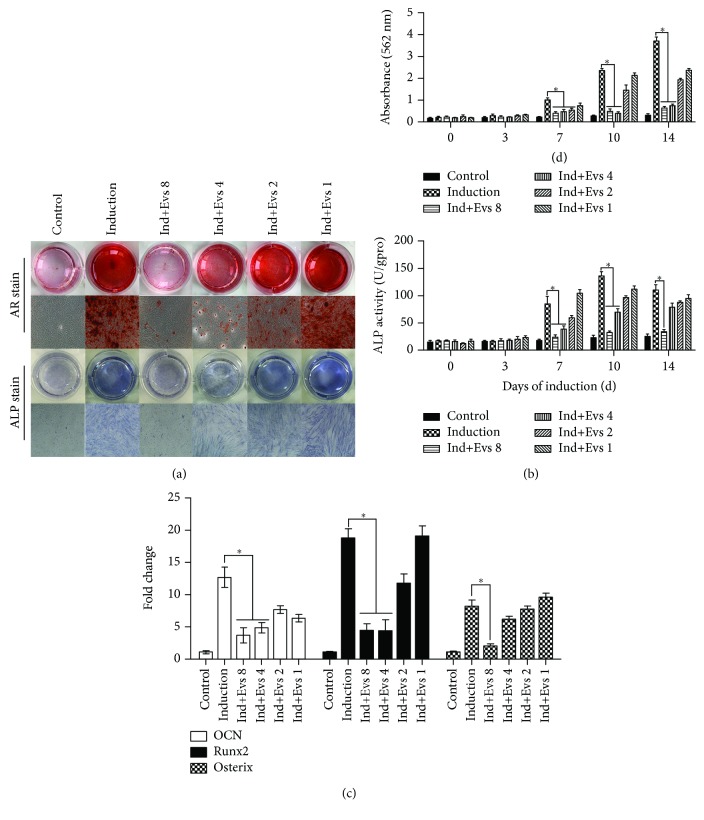
Plasma Evs suppress MSC osteogenic differentiation. Different concentrations of plasma Evs were added to the osteogenic differentiation medium in the Ind+Evs groups. Cells cultured in growth medium without induction served as the control group. (a) Photographs (top panels) and microscopy images (bottom panels) of ARS and ALP staining in representative samples from the control, induction, and Ind+Evs groups. Original magnification: ×40. (b) The ARS staining in (a) was quantified by monitoring the absorbance at 562 nm. The ALP activity in (a) was presented as units per gram of protein per 15 min. (c) Expression of osteoblast markers. The values represent the fold change in the gene expression relative to controls. Data obtained from six to nine samples per group are expressed as mean ± SE. ^∗^ *p* < 0.05 for the comparison between groups.

ARS staining and quantification were performed on the 14^th^ day after osteogenic induction to investigate the inhibitory effects of plasma Evs. According to the ARS staining (Figure 4(a)) and quantification (Figure 4(b)) results, plasma Evs significantly suppressed MSC osteogenic differentiation. Compared with the induction group, osteogenesis was markedly inhibited in the Ind+Evs 8 group (plasma Evs concentration: 12 × 10^8^ particles/mL) and the level of inhibition was proportional to the concentration of plasma Evs. As the plasma Evs concentration decreased (Ind+Evs 4: 6 × 10^8^ particles/mL, Ind+Evs 2: 3 × 10^8^ particles/mL, and Ind+Evs 1: 1.5 × 10^8^ particles/mL), the osteogenic differentiation and function of MSCs gradually recovered.

We extracted proteins from MSCs on days 0, 3, 7, 10, and 14 after osteogenic induction and observed peak ALP activity on the 10^th^ or 14^th^ days that decreased thereafter (Figure 4(b)). The results indicated significantly decreased ALP staining and activity in the Ind+Evs 8 group compared with the induction group. Therefore, we analysed three critical genes involved in osteogenic signalling pathways on day 10 of induction: OCN, Runx2, and Osterix. The expression of the OCN and Runx2 genes was reduced in the Ind+Evs 8 and Ind+Evs 4 groups, but the expression of the Osterix gene was decreased only in the Ind+Evs 8 group (Figure 4(c)). Based on the ARS staining, ALP activity, and expression of relevant downstream genes, plasma Evs inhibit the osteogenic differentiation and function of MSCs.

### 3.4. Plasma Evs Suppress MSC Osteogenic Differentiation by Regulating the Phosphorylation of Smad1/5/8

**Figure 5:**
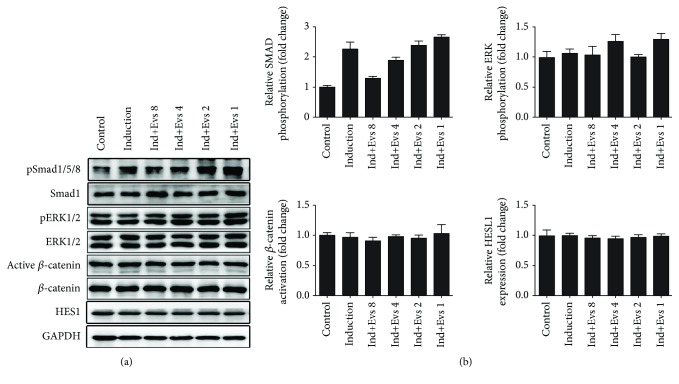
pSmad1/5/8 levels were downregulated by plasma Evs during osteogenic differentiation. (a, b) Activation of the Smad1/5/8, ERK1/2, *β*-catenin, and HES1 in MSCs was determined by Western blotting. GAPDH served as the loading control. Data from six to eight samples per group are presented as mean ± SE. ^∗^ *p* < 0.05 for the comparison between groups.

The osteogenic differentiation of MSCs is regulated by several signalling pathways, among which the Smad [24, 25], MAPK [26, 27], Wnt [28, 29], and Notch [30, 31] signal transduction pathways play major roles. Accordingly, we analysed the levels of the pSmad1/5/8, pERK1/2, active *β*-catenin, and HES1 proteins on the 10^th^ day after osteogenic induction. Levels of pSmad1/5/8 were markedly decreased in the Ind+Evs 8 and Ind+Evs 4 groups, and the level of inhibition positively correlated with the plasma Evs concentration (Figure 5(a)). The levels of pERK1/2, active *β*-catenin, and HES1 were not significantly different among the groups (Figure 5(b)). Therefore, plasma Evs inhibit osteogenic differentiation by reducing pSmad1/5/8 levels in MSCs.

### 3.5. Evs-Derived AHSG Is Involved in Suppressing MSC Osteogenic Differentiation

**Figure 6:**
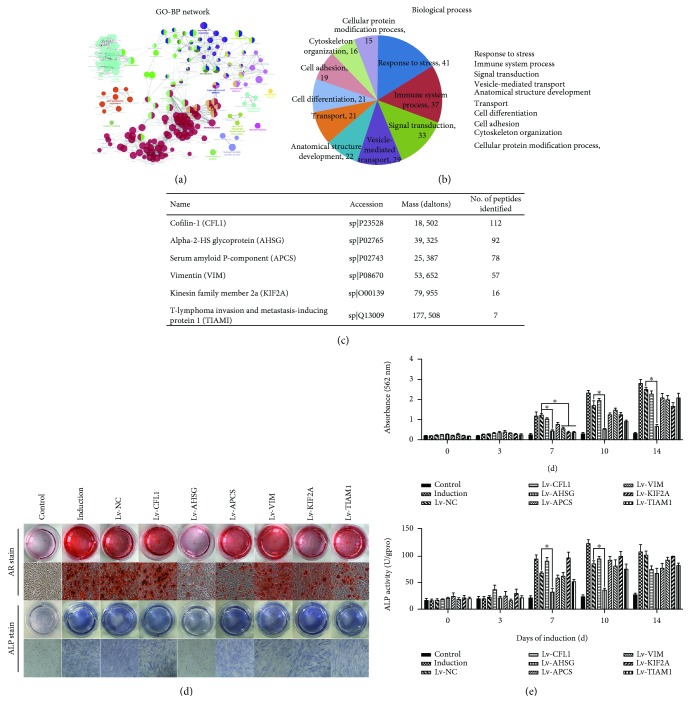
Identification and analysis of the plasma Evs proteome. Cells cultured in growth medium without induction served as the control group. Lentiviral vectors expressing CFL1, AHSG, APCs, VIM, KIF2A, and TIAM1 were added to the osteogenic differentiation medium in the Ind groups. (a) GO assignment of proteins in plasma Evs according to their assigned fraction and biological function. (b) Biological process. (c) No. of peptides identified. (d) Photographs (top panels) and microscopy images (bottom panels) of ARS and ALP staining in representative samples from the control, induction, and Ind+Lv groups. Original magnification: ×40. (e) The ARS staining in (d) was quantified by monitoring the absorbance at 562 nm. The ALP activity in (d) is presented as units per gram of protein per 15 min. Data were calculated from six to nine samples per group and are presented as mean ± SE.

We analysed the proteome of plasma Evs using mass spectrometry and identified 571 proteins. Ultimately, 21 proteins that are closely associated with cell differentiation were identified by GO analysis (Figures 6(a) and 6(b)). According to the confidence of their peptides, 6 identified proteins expressed at different levels were selected for further study: CFL1, AHSG, APCs, VIM, KIF2A, and TIAM1 (Figure 6(c)). We constructed lentiviral expression vectors to explore the roles of CFL1, AHSG, APCs, VIM, KIF2A, and TIAM1 in the osteogenic differentiation of MSCs. Cells were transduced with lentiviral vectors. According to the ARS and ALP staining, only Lv-AHSG inhibited the osteogenic differentiation of MSCs in the induction+Lv group (Figures 6(d) and 6(e)).

### 3.6. Reversal of pSmad1/5/8 Levels

**Figure 7:**
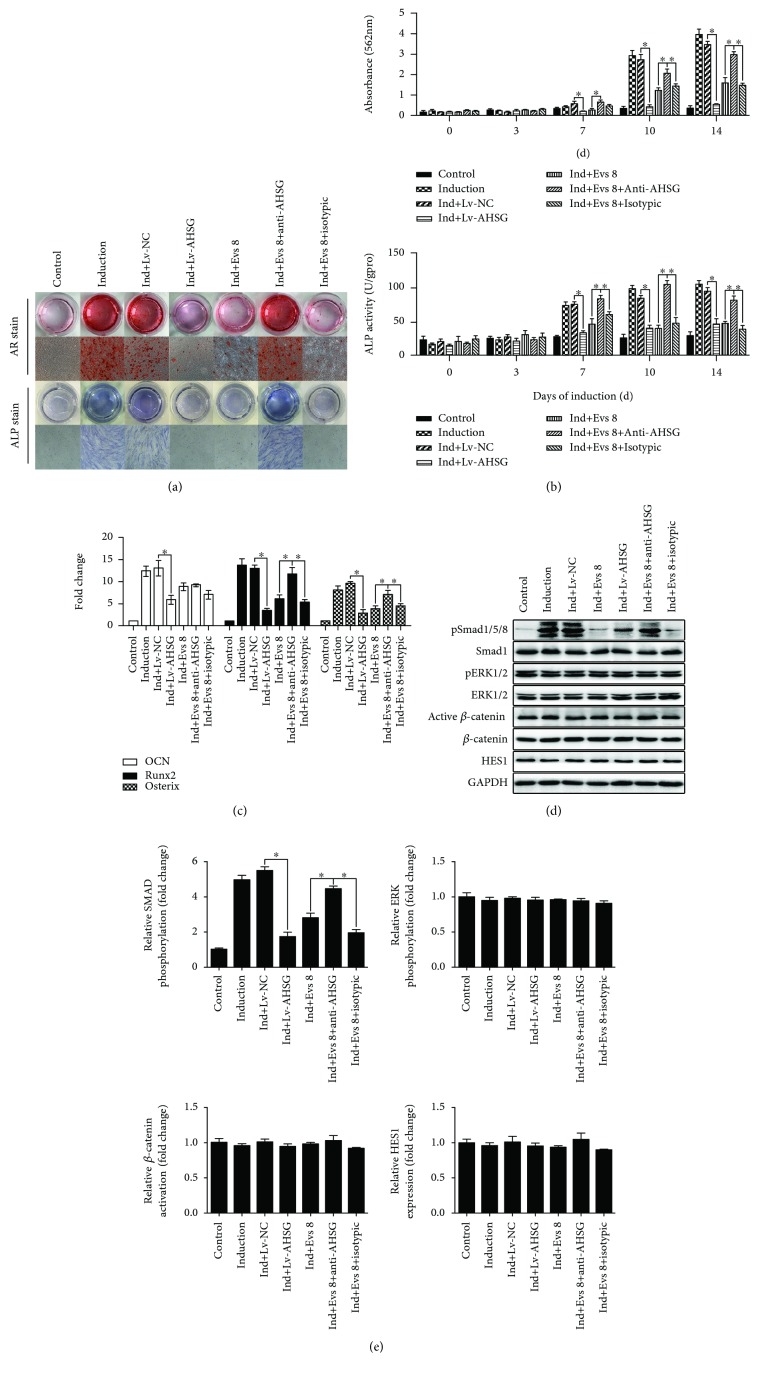
AHSG regulates pSmad1/5/8 levels in MSCs. Lv-AHSG and anti-AHSG were added to the osteogenic differentiation medium. Cells cultured in growth medium without induction served as the control group. (a) Photographs (top panels) and microscopy images (bottom panels) of ARS and ALP staining in representative samples from the control, induction, Ind+Lv-NC, Ind+Lv-AHSG, Ind+Evs 8, Ind+Evs 8+anti-AHSG, and Ind+Evs 8+isotypic groups. Original magnification: ×40. (b) ARS staining in (a) was quantified by monitoring the absorbance at 562 nm. ALP activity in (a) is presented as units per gram of protein per 15 min. (c) Expression of osteoblast markers. Values represent the fold change in gene expression relative to controls. (d, e) Levels of activated Smad1/5/8, ERK1/2, *β*-catenin, and HES1 in MSCs were determined by Western blotting. GAPDH served as the loading control. Data from four to six samples per group are presented as mean ± SE. ^∗^ *p* < 0.05 for the comparison between groups.

MSCs were treated with Lv-AHSG and a neutralizing antibody to investigate the inhibitory effects of AHSG. Based on the ARS staining, Lv-AHSG significantly inhibited the osteogenic differentiation of MSCs compared with the Lv-NC group (Figures 7(a) and 7(b)). Furthermore, the anti-AHSG antibody reversed the inhibitory effect of Evs 8 on the osteogenic differentiation of MSCs. Proteins were extracted from MSCs on days 0, 3, 7, 10, and 14, and ALP activity peaked on the 10^th^ or 14^th^ days in each group. Compared with the Ind+Lv-NC group, the Ind+Lv-AHSG group showed a noticeable decrease in ALP activity, but the Ind+Evs 8+anti-AHSG group showed a noticeable increase compared with the Ind+Evs 8 group and Ind+Evs 8+isotypic group.

We detected OCN, Runx2, and Osterix expression in MSCs on day 10 and found that compared with the Ind+Lv-NC group, the Ind+Lv-AHSG group showed an obvious decrease in OCN, Runx,2 and Osterix expression. The Ind+Evs 8+anti-AHSG group exhibited a marked increase in Runx2 and Osterix expression compared with the Ind+Evs 8 and Ind+Evs 8+isotypic groups. (Figure 7(c)). According to the ARS and ALP staining and the expression of crucial downstream genes related to osteogenesis, plasma Evs repress the osteogenic differentiation of MSCs through AHSG and that an anti-AHSG antibody reverses this inhibitory effect.

Based on the aforementioned results, plasma Evs repress the osteogenic differentiation of MSCs by decreasing pSmad1/5/8 levels. Therefore, we detected the levels of pSmad1/5/8, pERK1/2, active *β*-catenin, and HES1 in MSCs on 10^th^ day of osteogenic differentiation after treatment with Lv-AHSG and anti-AHSG. The induction group exhibited higher pSmad1/5/8 levels than the Ind+Lv-AHSG and Ind+Evs 8 groups (Figures 7(d) and 7(e)). In addition, pSmad1/5/8 levels were increased in the Ind+Evs 8+anti-AHSG group compared with the I Ind+Evs 8 and Ind+Evs 8+isotypic groups. Meanwhile, pERK1/2, active *β*-catenin, and HES1 levels were not significantly changed. Thus, AHSG in plasma Evs decreases pSmad1/5/8 levels in MSCs.

## 4. Discussion

Bone is a living tissue that is continually degraded and replaced. During the process of bone remodelling, environmental regulation is accomplished through the presentation of stimuli to bone cells. MSCs are the main effectors that regulate bone metabolism. The proliferative activity and functional status of MSCs directly reflect the various processes of bone remodelling in response to different environmental stimuli [32–34]. Plasma Evs, an essential component of the microenvironment, are secreted by various types of cells and play important roles in cellular communication. Using a fluorescence microscope, we observed that PKH26-labelled Evs were taken into the cytoplasm of MSCs within 8 h and that uptake increased over time. Plasma Evs contain diverse functional proteins, mRNAs, miRNAs, and DNA fragments, through which they influence the proliferation, differentiation, and function of surrounding cells. Compared with Evs secreted by a certain type of purified cell, the plasma Evs evaluated in our study enabled us to consider the problem from a holistic standpoint, and the results are therefore of greater significance. Because MSCs reside in a complex microenvironment that contains Evs from various sources in vivo, they receive different stimuli that eventually result in their differentiation and functional alterations. Our study is the first to describe the role of plasma Evs in repressing the osteogenic differentiation of MSCs.

We analysed the protein composition of plasma Evs and ultimately 6 identified proteins that were selected for further study. Then, we treated cells with lentiviral expression vectors to explore the roles of these proteins in the osteogenic differentiation of MSCs. According to the ARS and ALP staining, only Lv-AHSG inhibited the osteogenic differentiation of MSCs. AHSG plays a role in physiological and pathological mineralization [35, 36]. In vitro, AHSG prevents the uncontrolled mineralization of vascular smooth muscle cells by inhibiting mineral precipitation in intracellular vesicles [37]. Moreover, AHSG prevents calcification by buffering excess extracellular minerals and mediating their clearance in the form of calciprotein particles (CPPs) [38]. Based on the results from animal studies, AHSG inhibits ectopic calcification and regulates matrix mineralization in rat calvarial osteoblasts [39, 40]. Therefore, we speculated that as essential components of the extracellular microenvironment of MSCs, plasma Evs are endocytosed by MSCs and subsequently prevent calcification via AHSG-mediated mineralization.

The osteogenic differentiation of MSCs is affected by multiple signalling pathways, including the Smad [24, 25], MAPK [26, 27], Wnt [28, 29], and Notch [30, 31] pathways, which have important regulatory roles. These pathways interact to form an interconnected network that collaboratively regulates the osteogenic differentiation of MSCs and maintains bone homeostasis. According to our experimental results, treatment of MSCs with different concentrations of Evs did not significantly alter the expression of crucial proteins involved in the MAPK (ERK1/2 and pERK1/2), Wnt (*β*-catenin and active *β*-catenin), and Notch (HES1) signalling pathways. However, the levels of pSmad1/5/8 decreased as the concentration of Evs increased and were positively correlated with ALP activity and ARS staining. In order to distinguish the BMP/Smad and TGF*β*/Smad signalling pathways, we also detected the protein expression levels of pSmad2/3. The results showed that pSmad2/3 levels were not significantly different among the groups (Supplementary Figure 1). Thus, plasma Evs decrease pSmad1/5/8 levels in MSCs and further inhibit the osteogenic differentiation of MSCs.

According to our experimental results, the inhibitory effect on osteogenic differentiation of Evs was reversed following the addition of anti-AHSG antibodies. The levels of pSmad1/5/8 were increased in the Ind+Evs 8+anti-AHSG group compared with the Ind+Evs 8 and Ind+Evs 8+isotypic group. Meanwhile, pERK1/2, active *β*-catenin, and HES1 levels were not significantly changed. We also detected the protein expressions of TGF*β*1 and BMP2. But compare with the Ind+Evs 8 and Ind+Evs 8+isotypic group, the levels of TGF*β*1 and BMP2 in the Ind+Evs 8+anti-AHSG group were not significantly changed (Supplementary Figure 2). In a search for endogenous transforming growth factor-beta (TGF*β*) receptor antagonists, sequence homology was found between TGF*β* receptor type II and AHSG [15]. AHSG blocks osteogenic signalling pathways by binding directly to TGF*β* and with greater affinity to the TGF*β*-related BMPs, thereby inhibiting angiosteosis and the osteogenesis differentiation of MSCs [16]. We speculate that plasma Evs repress osteogenic differentiation of MSCs through AHSG-mediated competitive inhibition of BMP2 binding to its receptor, which reduced pSmad1/5/8 levels. However, anti-AHSG antibodies did not completely rescue MSC osteogenic differentiation to the same level as the induction group. Other proteins, miRNAs or lncRNAs in addition to AHSG might also contribute to the repressive effect of plasma Evs on osteogenesis.

Based on our results, AHSG in plasma Evs may have potential in the treatment and prevention of some metabolic bone diseases. AHSG expression is decreased in patients with ankylosing spondylitis, which may be one reason for pathological osteogenesis [41]. Strategies designed to increase the plasma Evs content of AHSG in patients with AS could diminish the enhanced osteogenesis. In addition, studies aiming to investigate whether the ossification of the posterior longitudinal ligament and other metabolic bone diseases are related to the plasma Evs content of AHSG would be worthwhile. Over the past decade, Evs have emerged as key cell-free tools for the treatment of a range of pathologies, including cancer, myocardial infarction, and inflammatory diseases [42]. Similar to autoimmunocyte therapy, modifying autologous Evs with target proteins and reintroducing them into the body will become a good method for decreasing immunological rejection.

In addition to our discoveries, many issues remain to be addressed. First, although plasma Evs repressed MSC osteogenic differentiation, we failed to identify the specific cell type that produced these functional Evs, making the differences in signal transduction patterns between two cell types impossible to elucidate. Second, although plasma Evs are superior to Evs produced by a specific type of cells at mimicking the internal environment, they constitute only a small part of whole plasma and do not completely reflect and represent the complicated conditions in the extracellular matrix of the human body. Based on the results from our preliminary experiments, Evs-free plasma and complete human plasma promoted the osteogenic differentiation of MSCs (Supplementary Figure 3), whereas plasma Evs notably repressed the same process. Third, we examined only the effect of plasma Evs containing AHSG on MSC differentiation towards the osteogenic lineage. We did not examine the effects of plasma Evs on the chondrogenic and adipogenic differentiation of MSCs, a topic worthy of further study. Finally, many animal models with AHSG defects have been established [43], and further studies are needed to ensure that the results of the animal experiments are consistent with the findings from our cell-based study.

## 5. Conclusions

In summary, our research identifies plasma Evs as novel regulators of MSC osteogenic differentiation. AHSG in plasma Evs inhibits the osteogenic differentiation of MSCs by decreasing pSmad1/5/8 levels.

## Data Availability

The data used to support the findings of this study are available from the corresponding author upon request.

## Supplementary Materials

Supplementary Materials Supplementary Figure 1: the levels of pSmad2/3 were not significantly different among the groups. (a, b) Phosphorylation of Smad2/3 in MSCs, as determined by Western blotting. GAPDH served as the loading control. Data from five samples per group are presented as mean ± SE. Supplementary Figure 2: the levels of TGF*β*1 and BMP2 in the Ind+Evs 8+anti-AHSG group were not significantly different among the groups. Lv-AHSG and anti-AHSG were added to the osteogenic differentiation medium. Cells cultured in growth medium without induction served as the control group. (a–c) Levels of TGF*β*1 and BMP2 in MSCs were determined by Western blotting. GAPDH served as the loading control. Data from four to six samples per group are presented as mean ± SE. Supplementary Figure 3: plasma Evs suppress MSC osteogenic differentiation, whereas Evs-free plasma and complete human plasma promoted the osteogenic differentiation of MSCs. Cells cultured in growth medium without induction served as the control group. (a) Photographs (top panels) and microscopy images (bottom panels) of ARS staining in representative samples from the control, induction, Ind+Evs 8, Ind+Evs-free plasma, and Ind+plasma groups. Original magnification: ×40. (b) The ARS staining in (a) was quantified by monitoring the absorbance at 562 nm. Data obtained from three to five samples per group are expressed as mean ± SE. ^∗^ *p* < 0.05 for the comparison between groups.
