# Supplementary material for: α2-HS Glycoprotein in Plasma Extracellular Vesicles Inhibits the Osteogenic Differentiation of Human Mesenchymal Stromal Cells In Vitro
Source: Stem Cells Int. 2019 Feb 7;2019:7246479. doi: 10.1155/2019/7246479 (PMC6383392; doi:10.1155/2019/7246479)
Supplement: Supplementary Materials — Supplementary Figure 1: the levels of pSmad2/3 were not significantly different among the groups. (a, b) Phosphorylation of Smad2/3 in MSCs, as determined by Western blotting. GAPDH served as the loading control. Data from five samples per group are presented as mean ± SE. Supplementary Figure 2: the levels of TGFβ1 and BMP2 in the Ind+Evs 8+anti-AHSG group were not significantly different among the groups. Lv-AHSG and anti-AHSG were added to the osteogenic differentiation medium. Cells cultured in growth medium without induction served as the control group. (a–c) Levels of TGFβ1 and BMP2 in MSCs were determined by Western blotting. GAPDH served as the loading control. Data from four to six samples per group are presented as mean ± SE. Supplementary Figure 3: plasma Evs suppress MSC osteogenic differentiation, whereas Evs-free plasma and complete human plasma promoted the osteogenic differentiation of MSCs. Cells cultured in growth medium without induction served as the control group. (a) Photographs (top panels) and microscopy images (bottom panels) of ARS staining in representative samples from the control, induction, Ind+Evs 8, Ind+Evs-free plasma, and Ind+plasma groups. Original magnification: ×40. (b) The ARS staining in (a) was quantified by monitoring the absorbance at 562 nm. Data obtained from three to five samples per group are expressed as mean ± SE. ∗ p < 0.05 for the comparison between groups. [file 7246479.f1.pdf]

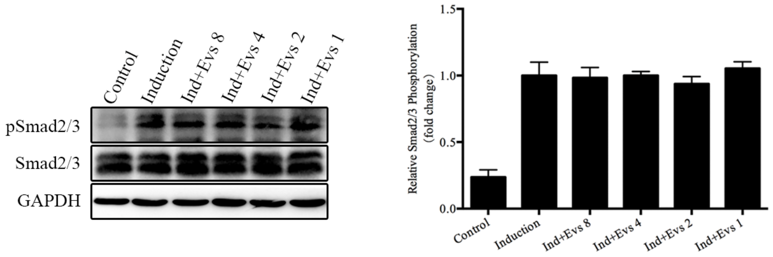

(a) (b)

Supplementary Figure 1: The levels of pSmad2/3 were not significantly different among the groups. (a-b) Phosphorylation of Smad2/3 in MSCs, as determined by Western blotting. GAPDH served as the loading control. Data from five samples per group are presented as mean ± SE.

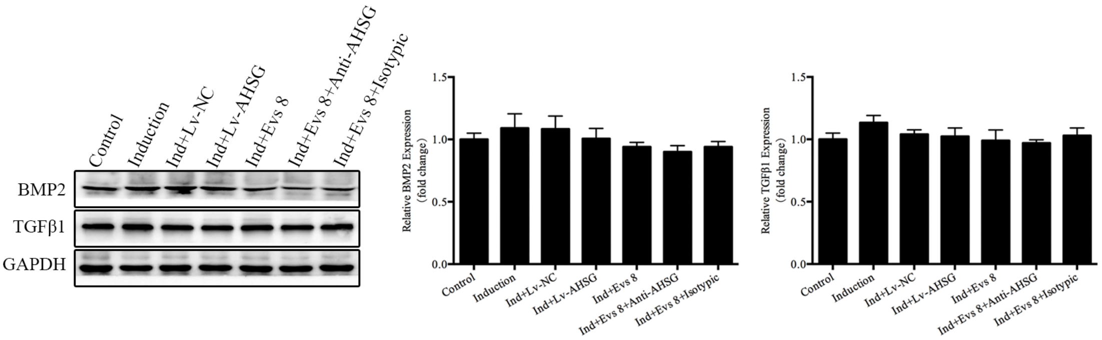

(a) (b) (c)

Supplementary Figure 2: The levels of TGFβ1 and BMP2 in Ind+Evs 8+Anti-AHSG group were not significantly different among the groups. Lv-AHSG and Anti-AHSG were added to the osteogenic differentiation medium. Cells cultured in growth medium without induction served as the Control group. (a-c) Levels of TGFβ1 and BMP2 in MSCs were determined by Western blotting. GAPDH served as the loading control. Data from four to six samples per group are presented as mean ± SE.

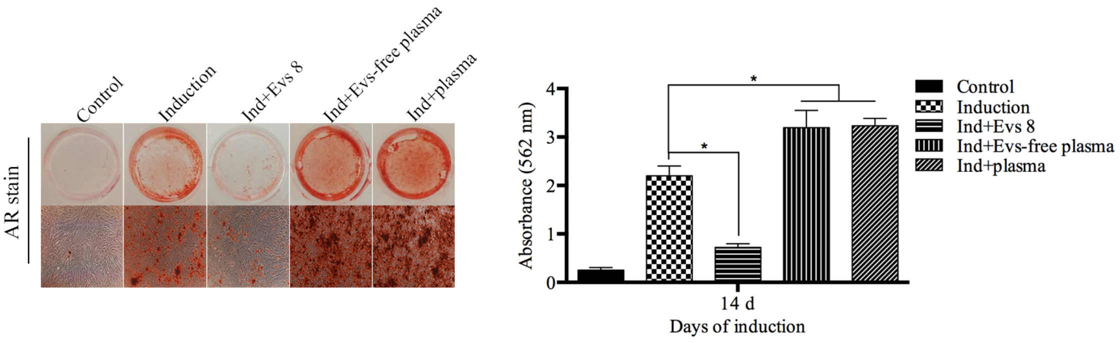

(a) (b)

Supplementary Figure 3: Plasma Evs suppress MSCs osteogenic differentiation, whereas Evs-free plasma and complete human plasma promoted the osteogenic differentiation of MSCs. Cells cultured in growth medium without induction served as the Control group. (a) Photographs (top panels) and microscopy images (bottom panels) of ARS staining in representative samples from the Control, Induction, Ind+Evs 8, Ind+Evs-free plasma, and Ind+plasma groups. Original magnification: ×40. (b) The ARS staining in (a) was quantified by monitoring the absorbance at 562 nm. Data obtained from three to five samples per group are expressed as mean ± SE. *p<0.05 for the comparison between groups.
